# The Use of Potent Populations of Expanded Fetal Human Placental Stromal Cells for the Treatment of Dextran Sodium Sulfate-Induced Colitis in a Mouse Model

**DOI:** 10.3390/ijms26073222

**Published:** 2025-03-30

**Authors:** Raphael Gorodetsky, Astar Lazmi Hailu, Evgenia Volinsky, Boaz Adani, Orit Pappo, Eran Israeli

**Affiliations:** 1 Laboratory of Biotechnology and Radiobiology, Sharett Institute of Oncology, Hadassah-Hebrew University Medical Center, Jerusalem 9112001, Israel; astarlazmi@gmail.com (A.L.H.); evgenia.volinsky@gmail.com (E.V.);; 2 Department of Pathology, Hadassah-Hebrew University Medical Center, Jerusalem 9112001, Israel; lorit@hadassah.org.il; 3 Department of Gastroenterology, Hadassah-Hebrew University Medical Center, Jerusalem 9112001, Israel; erani@wmc.gov.il

**Keywords:** expanded fetal human placental stromal cells (f-hPSCs), inflammatory bowel disease (IBD), cell therapy, C3H mouse model, adult cell therapy

## Abstract

Inflammatory Bowel Disease (IBD) is a multifactorial gastrointestinal condition encompassing two major forms of intestinal inflammation: Crohn’s disease (CD) and ulcerative colitis (UC). Both conditions are linked to auto-inflammatory reactions and genetic predispositions. Various drug therapies and biological treatments proposed to reduce IBD-associated inflammation. We induced IBD in a mouse model by stimulating bowel inflammation with an oral dextran sodium sulfate (DSS) beverage. Our novel cell therapy approach for IBD involves intramuscular (IM) and intraperitoneal (IP) delivery of non-matched, expanded, potent xenogeneic fetal human mesenchymal stromal cells (f-hPSCs) in 2 × 10^6^ cell injections. This cell therapy has already been shown previously to induce pro-regenerative and anti-inflammatory effects in different systemic and local disorders, where the injected f-hPSCs were shown to respond to the stress of the host and secrete the adequate secretome in response to this stress. In the current study, the IP-injected f-hPSCs treatment of the DSS-induced IBD enhanced the regenerative processes of the damaged bowel and reduced the inflammatory process. This was associated with rapid regain of the mice’s weight and a decrease in inflammation-associated parameters, such as colon edema, bowel shortening, and a threefold increase in bowel mass, as estimated by increased colon weight and reduced length. This ratio best emphasized the induced inflammatory response associated with the decrease in the inflamed colon length with an increase in its mass. Although IM f-hPSCs delivery was somehow effective by a few parameters, the IP delivery produced a superior response. The IP f-hPSCs treated mice lost only ~15% of their weight at the peak of the IBD effect, compared to ~25% in untreated mice. A reduction in the inflammatory response of the gut was also indicated by a decrease in neutrophil infiltration, as assayed by a myeloperoxidase (MPO) assay. Additionally, a significant improvement in the histological score of the gut and faster recovery to 90% of its original size was observed. These findings suggest that f-hPSC treatments could serve as an effective and safe anti-inflammatory and pro-regenerative treatment for IBD.

## 1. Introduction

Ulcerative colitis and Crohn’s disease (CD) are distinct manifestations of Inflammatory Bowel Disease (IBD), both involving inflammation along the lining of the gastrointestinal tract, particularly the large intestine (colon) and rectum. These multifactorial disorders are commonly associated with a disrupted interaction between the host immune system and the commensal microflora [1,2,3]. This interaction can include an overreaction to non-virulent luminal antigens, leading to mucosal inflammation. This disruption may include an overreaction to non-virulent gut viruses, bacteria, certain foods, and other luminal antigens, ultimately leading to gut inflammation [1,2].

Many treatments for CD are based on novel biological therapies, such as monoclonal antibodies that target pro-inflammatory cytokines in the affected organ and modulate cellular immune responses [3,4,5,6,7].

Cell-based therapy approaches for the treatment of IBD were also proposed, such as intravenous administration of adult bone-marrow-derived mesenchymal stromal (BM-MSCs) and mesenchymal stem/stromal cells from other sources, with different routes of the cell delivery [7,8,9,10]. However, the exact mechanism of the proposed benefits of these cell-based therapies has not been fully understood [8]. MSC treatments in animal and human studies to indirectly alleviate CD are generally based on the expected indirect modulation of the inflammatory processes, to induce significant disease remission with notable mucosal healing [7]. A possible repair of the damaged tissues by integration of the implanted cells in the tissue has not been demonstrated and there has been no clear evidence that locally injected stem cells differentiate to form building blocks of damaged tissues in the gastrointestinal tract. In MSC therapy, the effect appeared to be associated with the pro-regenerative secretome of the different cell sources tested. Local IBD treatments based on MSCs in related studies showed that the cells indirectly helped to control the inflammatory processes, leading to modest remission of the disease, including notable mucosal healing [8,9,10,11,12]. Studies on cell-based therapies for different disorders by their injection into the blood stream have shown that therapeutic cells rarely target the damaged tissues directly. Rather, the injected cells are often trapped in micro-vessels, primarily in the lungs, and their observed effects do not appear to stem from their “stemness” [13,14,15]. This suggests their indirect influence, possibly through the induction of the secretion of anti-inflammatory cytokines, pro-regenerative growth factors, and immune-tolerance-inducing agents, both in soluble form or in exosomes [10,13,14,15,16,17].

MSCs from various sources may differ in their anti-inflammatory and pro-regenerative effect. In the current study, we used cells isolated specifically from fetal human placental stem cells (f-hPSCs). The remote delivery of f-hPSCs, isolated and expanded from the connective tissues of the chorionic plate of the full-term fetus, was found to be highly effective in the induction of pro-regenerative processes, in comparison to the similarly isolated and expanded maternal placental stromal cells [13,16,17,18,19,20,21,22,23,24,25,26]. We propose that the very stable fully differentiated phenotype of f-hPSCs seems to have a significant advantage over the unstable differentiation state of expanded BM-MSCs, with stem cell properties with a risk of possible non-desired trans-transformation to other cell phenotypes following their delivery.

Flow cytometry (FACS) of f-hPSCs surface protein profile demonstrates typical surface markers of MSCs, with the positive expression of CD29 and CD90, and somehow lower expression of CD105 [16,19,20]. As mesodermal cells, the f-hPSCs are negative to typical surface markers of hematopoietic cells, such as CD45, CD19, and CD11b, and the endothelial marker CD34, as well as low expression of HLA-DR and HLA-G, which may contribute to the immuno-tolerance towards these cells in allo/xenogeneic treatments. The f-hPSCs were also reported to express CD146 and CD166 as well as CD276, which are not expressed in most other types of MSCs. These markers could also be related to the relative tolerance towards this injected cell [20,21,22,23].

We suggest that upon the remote or local delivery of f-hPSCs, they may sense stress signals from damaged organs, with subsequent secretion of pro-regeneration secretome that can reach the affected tissues. This was also shown in different experimental works with other stromal cell-based CD therapies [10,12,15,19]. The higher potency of f-hPSCs may be associated with their apparent higher sensitivity to the systemically released stress signals from the damaged tissues, which induce them to release higher titers of modifying factors to address the relevant tissue damages [19,20,24].

There is a major straightforward advantage of therapies based on the injection of the xenogeneic or allogeneic non-matched f-hPSCs into vascularized tissues, in contrast to the delivery of the cell secretome only. As we have previously shown, the f-hPSCs can persist in their injection site for relatively prolonged periods of more than 6 days before their clearance. During that period, they seem to respond to the systemic host’s stress signals in the circulation. Subsequently, they are induced to continuously secrete for several days relevant factors that may promote the tissue recovery, before they are cleared away [16,17,19,20].

Different studies have proposed the delivery of various types of MSCs and other cell types to treat experimental IBD [11,27,28,29,30,31]. Some of them suggest the local injections of MSC-derived secretome for treating IBD, either in free form or encapsulated in protective slow-releasing matrices, may be more effective than remotely injecting active living cells. However, the remote injection of highly responsive cultured f-hPSCs into densely vascularized tissues, such as large muscles or the peritoneum, offers a significant advantage for a maximal prolonged systemic effect [16,17,28]. The response of the f-hPSCs to the systemically circulating stress signals can endure for several days, until the final slow clearance of the f-hPSCs, and the parallel decrease in the secretion of stress signals in the course of the healing of the affected tissues, as we have clearly shown in mitigating severe acute radiation-induced bone marrow damages [19].

## 2. Results

### 2.1. Characterization of the f-hPSCs

**Figure 1:**
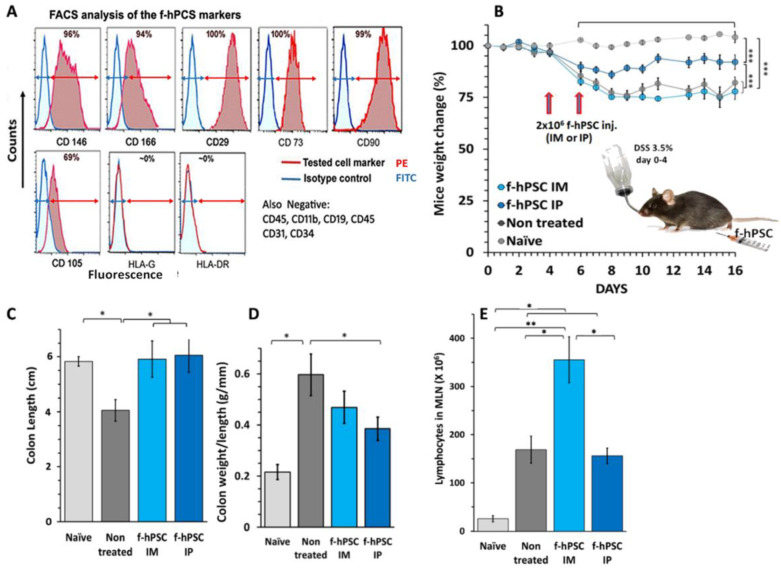
DSS-induced colitis experiment and f-hPSCs treatment. (**A**) FACS analysis of the cell surface markers of the isolated and expanded f-hPSCs; the typical mesenchymal markers gating was determined for each of the markers tested (range shown by red arrow range), to be positive above the 95% range of the readings of isotypes controls (range shown by blue arrows). (**B**) Four experimental arms were tested: 3 groups of mice were exposed to 3.5% DSS in drinking water for 4 days. f-hPSCs were injected either IM or IP into the adequate treated groups on days 4 and 6. (**C**) At the termination of the experiment, the colon was excised, weighed and its length measured. (**D**) The records of the colon mass/length ratio at the end of the experiment. (**E**) Lymphocyte counts in the MLN cells at the end of the experiment. The bars above the columns indicate the significance of the difference between the tested groups. Light gray fill represents naïve controls, dark gray represents the non-treated DSS exposed group and light blue and dark blue represent IM and IV f-hPSCs treated DSS exposed mice, respectively. (* = *p* < 0.05, ** = *p* < 0.01, *** = *p* < 0.005). Except for the group of “Naïve” mice, all other groups were exposed to DSS.

The f-hPSCs used were isolated from the donated human fetal placenta tissue of male offspring following a planned caesarian section, donated with the full informed consent of both parents, as previously described [17,18,21]. The cell source was verified as the mesenchymal tissues of only the fetal placenta by their X-Y karyotype. FACS analysis of cell surface markers of the expanded f-hPSCs confirmed their typical mesenchymal stromal cells (MSC) markers. As previously reported and verified in the current work, FACS analyses verified that the cells had positive expression mesenchymal markers CD29 and CD90, and were partially positive for CD105 expression, a marker prominently expressed in many other types of mesenchymal stromal cells (Figure 1A). Additional markers positively expressed on f-hPSCs included CD146 as well as CD166, which are typically expressed in the chorion-derived hPSCs. Similar to stromal cells isolated from other mesenchymal tissues, the f-hPSCs were found negative for hematopoietic markers CD45, CD19, and CD11b. The expression of HLA-DR and HLA-G as well as the endothelial cell marker CD34 on the f-hPSCs were negative (Figure 1A).

### 2.2. DSS Induced IBD

The experimental setup for the induction of IBD in the mice is presented graphically in Figure 1B based on previously published protocols [32,33]. The three different groups tested were exposed to 3.5% DSS in their drinking water for four days. The nearly confluent cultured f-hPSCs were trypsinized before their injection, washed once, and counted, as previously described [17]. In each cell treatment, 2 × 10^6^ f-hPSCs were injected on days 4 and 6, either in one injection intra-peritoneally (IP) or divided into two injections to the muscles of both legs intramuscularly (IM). In the untreated group, only the medium solution with no cells was injected. During the follow-up, mice exposed to DSS experienced a very significant weight loss from day 8 onward to the end of the follow-up (Figure 1B, *p* < 0.005), but none of them in any of the experimental groups died during the DSS exposure period up to the end of the experiment. Still, those injected with f-hPSCs IP lost significantly less weight than the non-cell treated controls or the IM-injected mice (Figure 1B, *p* < 0.01). The DSS-exposed groups showed continuous weight loss between days 0–8 after the onset of IBD. The non-treated group and the group treated with IM f-hPSCs lost about 25% of their body weight, with no significant weight regain by the end of the 16 days of follow-up. In contrast, mice injected IP with f-hPSCs experienced significantly lower body weight loss by day 8 (approximately 15%, *p* < 0.005) for the whole period of the 16-day follow-up (Figure 1B).

### 2.3. The Influence of f-hPSCs Injection on the Mice Colon

On day 16, at the conclusion of the follow-up and mice euthanization, their colons were dissected, cleansed, and measured. Inflammatory colitis with extensive swelling due to an inflammatory process with significant colon shortening was observed in the groups of DSS-treated mice, only in the non-f-hPSCs treated group, in comparison to other arms tested (*p* < 0.05). In the f-hPSCs-treated DSS-exposed groups, no significant difference in colon length, relative to naïve controls, was observed (Figure 1C). However, the most notable changes in colon mass were demonstrated by the colon weight-to-length ratio (Figure 1D). As expected, the inflamed elevated mass per unit length of the shrinking colon seemed to increase in all groups of mice exposed to DSS and was most notable and significant in the non-treated group with a significant difference from controls (*p* < 0.05). Treatment with f-hPSCs reduced the mass per length increase in the inflamed DSS-induced colons significantly by approximately 30%, as compared to the non-cell-treated group (*p* < 0.05), though it was slightly higher, albeit not significantly different as compared to the naïve controls (Figure 1D).

### 2.4. The Influence of f-hPSCs Treatments on the Total Mesenteric Lymph Nodes (MLN) Cell Counts

The number of cells harvested from the MLN, consisting primarily of lymphocytes, showed a significant increase in all experimental groups (*p* < 0.05), which was most significant (*p* < 0.01) following DSS consumption, in comparison with the naïve controls (Figure 1E).

### 2.5. Effect of the DSS and f-hPSCs Treatments on the Peripheral Blood Cell Counts

**Figure 2:**
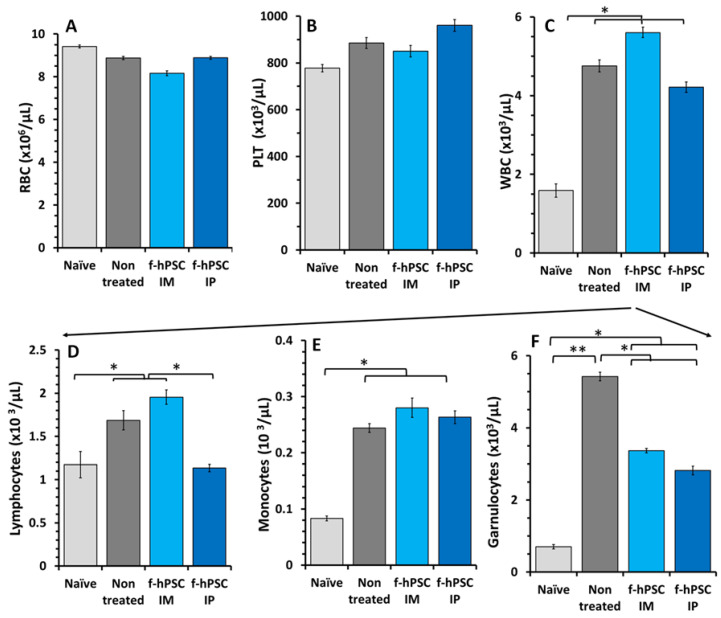
CBC at the end of the experiment. CBC values performed at the termination of the experiment for the different experimental groups tested are presented. Light gray fill represents naïve controls, dark gray represents the non-treated DSS exposed group, while light blue and dark blue represent IM and IV f-hPSCs treated DSS exposed mice, respectively. The results of RBC (**A**) PLT (**B**) and WBC are presented (**C**). The peripheral WBC was found significantly elevated in all the DSS-exposed mice. Differential leukocyte counting (highlighted by arrows) showed a major decrease in lymphocyte number in f-hPSCs DSS exposed IP treated mice (**D**). Similar significant elevations of monocyte counts were recorded in all DSS-exposed mice (**E**). The most apparent effect of the f-hPSCs treatment is indicated by a very significant reduction in the granulocyte counts in the f-hPSCs IP-treated mice (**F**). The bars above columns indicate the significance of the difference between the groups tested. (* *p* < 0.05, ** *p* < 0.01). Except for the mice group of Naïve, all other groups were exposed to DSS.

A small non-significant decrease in RBC counts was observed at the end of the experiment across all experimental arms of the DSS-exposed mice (Figure 2A), most likely due to the DSS-induced bleeding in the inflamed GI tract. However, no significant differences between the f-hPSCs-treated and non-treated mice were noted. Platelet counts were slightly and non-significantly elevated in all DSS-exposed mice relative to naïve controls and f-hPSCs-treated groups (Figure 2B). Total white blood cell (WBC) counts, indicative of significant inflammatory processes, increased significantly (*p* < 0.05) in all DSS-exposed groups, with slightly non-significant higher counts in the non-treated group, compared to the f-hPSCs IP-injected mice (Figure 2C). Similarly, lymphocyte counts were significantly elevated in both the non-treated and IM f-hPSCs-treated DSS-exposed mice (*p* < 0.05) (Figure 2D, while counts in the IP f-hPSCs-treated group were comparable to the naïve controls and similar to the non-DSS-treated group. Monocyte counts were elevated in all DSS-exposed mice relative to the naïve controls (Figure 2E). The differences in granulocyte counts between the groups were even more apparent, with the non-treated DSS-exposed group showing the significantly highest counts (*p* < 0.01), compared to less elevated, but still significant (*p* < 0.05) high counts in DSS-exposed mice treated with f-hPSCs via IM or IP injection (Figure 2F).

### 2.6. Colon Histology

**Figure 3:**
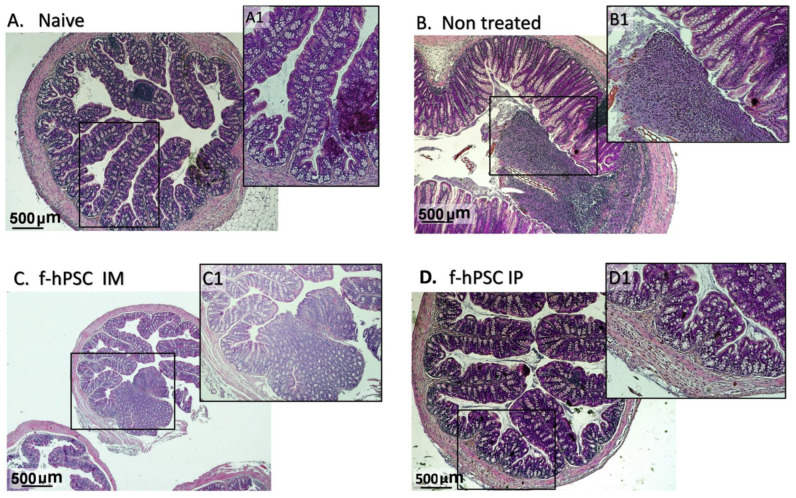
H&E stained Histology of the colon. Small colon samples were collected from the sacrificed mice and were processed in paraffin for histology. Representative H&E stained slides were photographed for each group tested (**A**–**D**) with magnification of a selected area (**A1**,**B1**,**C1**,**D1**). The histological sections were used for scoring the severity of the inflammatory response as presented in Figure 4A. The “Naïve” control mice (**A**) were not exposed to DSS, and (**B**–**D**) groups were mice exposed to DSS.

Histological sections of the colon performed on H&E colon paraffin sections of the different arms tested are presented in Figure 3A–D with further magnification of a representative region in each arm, marked by the detailed low magnification microphotographs enlarged from the black inner rectangle. A representative section of the colon of a mouse injected without cells is shown in Figure 3A. In the section of non-treated mice (Figure 3B), the crypts are seen as severely damaged or depleted and were replaced with inflammatory reactions. The histological colon sections of IM and IP f-hPSCs treated mice are shown in Figure 3C,D, respectively. Major regeneration processes are seen in the DSS-exposed mice treated with f-hPSCs injections, with the best effect, apparently, in those treated IPs.

**Figure 4:**
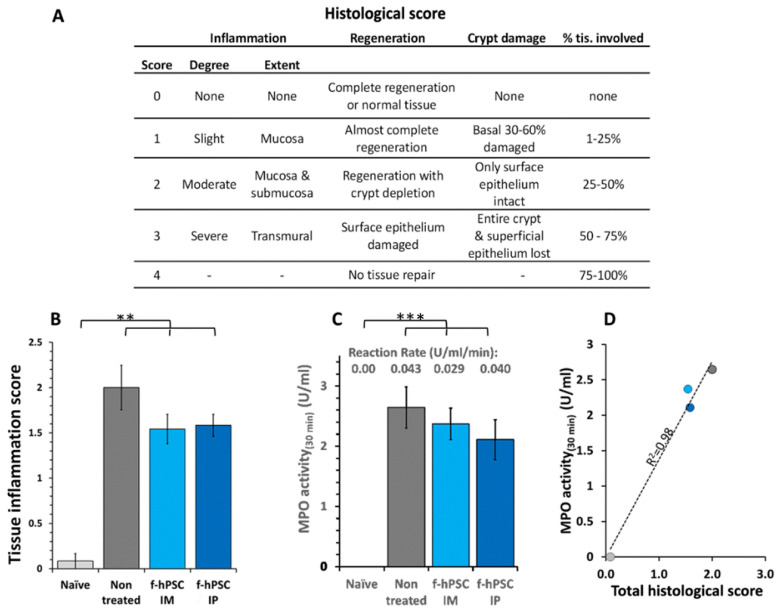
Tissue inflammation and regeneration. The records of the blind histological score for evaluation of tissue inflammation and regeneration parameters are shown in (**A**). The results of the MPO activity are shown in (**B**). The correlation between the severity of the inflammatory response as evaluated by MPO assay for infiltrated neutrophils is presented in (**C**). The bars above columns indicate significant differences between groups, where applicable, (** = *p* < 0.01, *** = *p* < 0.005). Except for the mice group of “Naïve”, all other groups were exposed to DSS. The ratio between the MPO activity and histological inflammation score in all groups tested is shown in (**D**). In all the graphs presented light gray fill in columns and data points represents naïve controls, dark gray represents the non-treated DSS exposed group, while light blue and dark blue represent IM and IV f-hPSCs treated DSS exposed mice, respectively.

The main histological parameters for objective, blinded scoring based on the assessment by a pathology expert, are presented in the table in Figure 4A, using a scale of 0–4. The parameters evaluated included the extent of regenerating tissue, the degree of crypt damage, and the percentage of tissue involved in the degeneration process. The score for inflammatory processes was found to be significantly highly elevated in all DSS-exposed groups (*p* < 0.01), with the highest values in non-treated mice (Figure 4B). The f-hPSCs-treated groups showed somewhat lower scores, although the differences were not statistically significant. The myeloperoxidase (MPO) assay reaction rate, shown in Figure 4C, was negligible in the naïve group and very significantly elevated in all the DSS-exposed mice (*p* < 0.001). The results are further substantiated by the very high correlation (r^2^ = 0.98) between the MPO activity and the histological score (Figure 4D).

### 2.7. MPO Assay for Neutrophils Activity

Another parameter associated with inflammation is the density of neutrophils in the colon as evaluated by the assay of MPO enzymatic reaction activity. A maximum reading was reached within 30 min. In all groups of mice exposed to DSS, the level of MPO in the colon at the end of the follow-up was increased significantly relative to the control group, indicating the recruitment of neutrophils to the inflamed colon tissues (Figure 4C). The MPO activity in the group IP injected with f-hPSCs was slightly but not statistically decreased (Figure 4C), with a high correlation (f = 0.98) between the MPO activity levels in the tissues and the histological score in the different groups tested (Figure 4D).

**Figure 5:**
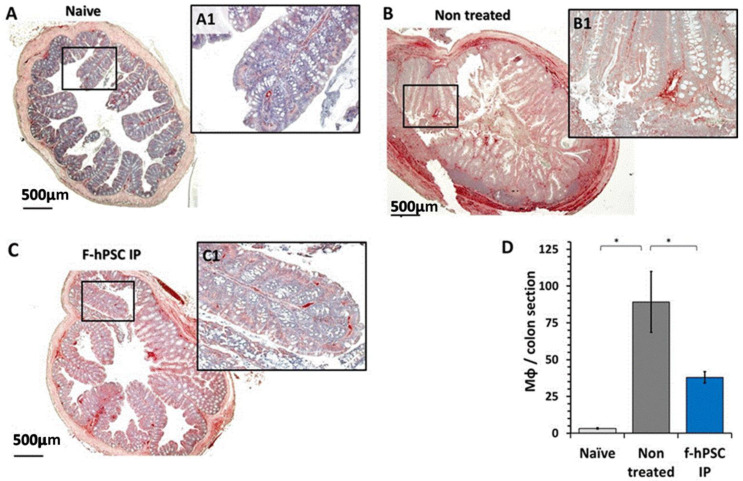
Mϕ infiltration to the crypts of the colon. Immuno-stained histology of Mϕ infiltration in the colon histological sections of the different groups was tested. The sections of the colon of all mice in different arms were immune-stained with CD68 (with hematoxylin background). (**A**–**C**) with magnification of a selected area marked as rectangle in the low magnifications micrographs (**A1**,**B1**,**C1**). Counts of Mϕ number per total tissue area in the immune-stained sections for naïve, non-treated, and IP f-hPSCs groups are shown (**D**) (* = *p* < 0.05). Except for the mice group of “Naïve”, all other groups were exposed to DSS. Light gray fill represents naïve controls, dark gray represents non-treated DSS exposed group, and dark blue represents IV f-hPSCs treated DSS exposed mice.

The inflammatory processes in the colon involved an increased Mϕ infiltration into the affected crypt tissues. The counting of the CD68 immune-stained Mϕ in the colon tissue sections area is shown in Figure 5A–C for the 3 main groups of interest with magnifications of a region of interest in each picture (A1, B1, and C1, respectively). Figure 5D shows the Mϕ density with a significant (*p* < 0.05) elevation in the prevalence of the Mϕ in the group of DSS exposed nontreated mice. In the f-hPSCs injected mice, the density of Mϕ in the tissue was significantly reduced (*p* < 0.05), towards the range of normal values.

In the control group (Figure 5A,A1), a fewer number of Mϕ are observed in the crypts, primarily located in the periphery. In contrast, in non-cell treated DSS exposed mice with inflamed colon, a high number of Mϕ are present in both the crypts and the submucosa (Figure 5B,B1). Following IP f-hPSCs treatment (Figure 5C,C1), a significant reduction (*p* > 0.05) in Mϕ density per gut circumference relative to untreated mice was observed, reaching almost their normal density (Figure 5D).

## 3. Discussion

Cell therapies with different types of potent/stem cells have been proposed as effective pro-regenerative therapy. In most of the related studies, the predominantly indirect, effect of the cells was found to be mediated through a vast release of different pro-regenerative and anti-inflammatory factors [34,35,36,37,38]. Due to the easily available source of these cells, PSCs were suggested as potentially active cells for different cell treatments [8,13,14,15,16,17,18,19,20,21,22,23,24,32,37,38,39]. In many of the previously reported studies on PSCs-based cell therapy, the cells were injected systemically by IV delivery, rather than by IP route, as we proposed here. Only in a few of these studies, the critical issue of the origin of the cells from the fetal tissue source of the placenta was referred to [8,17,18,19,20,35]. Others have suggested that the over-reaction of the immune system in the GI tract in CD is possibly mediated by activated CD4(+)-T cells lymphocytes under Th17 differentiating conditions, which has been proposed as the mechanism of induction of the undesired inflammatory reaction associated with IBD [34,36].

In most previous studies, no clear indication was given of the exact placental tissue source used. In such cases, the isolated stromal cells used should have probably derived from the maternal placental tissues, which is the predominant stromal cell population upon the isolation of the cells from the whole placenta. This may be a critical issue, as we previously demonstrated the therapeutic advantage and the higher potency of f-hPSCs isolated from only the fetal placental tissues [17,35].

The mechanism behind the enhanced activity of the f-hPSCs may be associated with the role of their tissue origin in the support of the growing fetus, supplying its essential needed factors and nutrients during its development and anti-inflammatory agents. The primary role of the tissues of the maternal placenta, which is not directly connected to the fetus’s blood system, appears to be the support and provision of general protection to the expansion of the growing allogeneic fetal placenta and the fetus.

Placental stromal cells, in general, and f-hPSCs particularly are fully differentiated stable phenotypes of mature cells and they are not “stem cells” by definition, though they are presented sometimes as such. Due to their negligible potential for trans-differentiation, they are not expected to serve as building blocks of the regenerating tissues. Moreover, no specific homing is expected following the systemic injection of these cells into tissues or different body spaces. Following their delivery into the tissues, the cells were shown to reside within the injected site until their slow clearance, with minimal rejection or complications [16]. During that time frame, these or other types of stromal cells, tend to respond to blood-born stress factors by releasing pro-regenerative immunomodulating and anti-inflammatory secretome [19,39,40,41,42,43]. We did not explore the option of f-hPSCs injection via IV administration, since IV-injected cells are expected to become trapped in the lung microvascular system during their first pass until their clearance, with possible related complications [15,16]. The f-hPSCs are derived exclusively from fetal placenta tissue and are expected to mediate highly efficient anti-inflammatory and pro-regenerative effects. The f-hPSCs, specifically, were shown to have negligible rejection rate for longer time intervals in the injected tissues, allowing the extended release of their secretome, before being cleared away [27,40,41,42,43,44,45].

The f-hPSCs isolated from the fetal placenta tissue were shown to express low levels of MHC class I antigens. They lack MHC class II on top of their CD276 expression, all these factors may contribute to the reduction in their immune rejection [20,44,45,46,47,48]. This unique expression profile allows these placental cells to confront immune detection and maternal-fetal tolerance during pregnancy, with their reported secretion of immune-regulatory factors, such as transforming growth factor-beta (TGF-β), indoleamine 2,3-dioxygenase (IDO), and interleukin-10 (IL-10), which may help suppress inflammatory responses, enhance immunotolerance and promote tissue repair [10,36,42,49].

The effect of inflammation of the gut was found to be associated with the increase in the colon mass associated with a decrease in its length. Each of these parameters and their ratio were previously proposed to serve as highly indicative of the inflammatory state of the gut [50,51,52]. In the current study, we showed that IP injection of f-hPSCs (and to a lesser degree also via IM administration) alleviated the inflammatory IBD induced by DSS, as expressed by prevention of colon shortening, and increase in its mass, reduction in the number of circulating granulocytes and significant improvement in most other inflammation-related parameters tested. These parameters were previously proposed for evaluation of the severity of its inflammation process. The reversal of these changes, as well as reduced MPO activity and, a decrease in the number of infiltrating Mϕ counts in the colons in the histological section were associated with improved histological scoring of the regenerative process and inflammation of the affected gut [53,54]. These effects of the f-hPSCs treatment were more significant than the earlier described results in a similar experimental setup treated locally with non-specific MSC secretome [28,30]. These results add more evidence to support our previously described findings on the indirect effect of f-hPSCs treatments in the induction of significant anti-inflammatory and pro-regenerative effects in other degenerative conditions [16,17,19,21,43]. In spite of our expectation that f-hPSCs may be active when injected into any remote vascularized site, our findings suggest that the intraperitoneal (IP) administration of f-hPSCs is a more effective anti-inflammatory and pro-regenerative treatment in IBD, as compared to intramuscular (IM) administration. Further detailed studies on the indirect mechanism of this pro-regenerative effect induced by the f-hPSCs and the contribution of the secreted growth factors and/or anti-inflammatory molecular free agents, or packed in exosomes, are of major interest to better understand this phenomenon [24,30,39,42,43,53,54,55].

So far, our findings indicate that f-hPSCs, cultured under properly regulated conditions, could serve as a safe and effective xenogeneic or allogeneic cell therapy for clinical application for treatment for alleviating IBD. Clinical studies have already examined the safety and tolerability of different PSC preparations, isolated from postpartum placenta and administered via IV injection with different doses of allogeneic cells. As previously shown, these cells were generally well tolerated, with only minor adverse effects. The need for corticosteroids to prevent rejection during the short interval activity of the cells following their injection seemed to be redundant, in view of their low allogeneic on one hand, low xenogeneic rejection, and the short time interval needed to exert their effect [10,17,19]. Phase 1b/2a study already established the safety and tolerability of 1.5–6 × 10^8^ injected placental cells in humans, associated with clinical improvement, and reduction in inflammatory markers, such as CRP, with no significant adverse event. Further studies on this potent cell therapy should be encouraged as a simple treatment of active IBD. We expect that such studies could further establish our findings that mesenchymal cells isolated from the fetal placental tissues and further expanded may be more potent and immuno-competent than other non-tissue-matched therapeutic cells.

In conclusion, the findings of this study underscore the potential of utilizing an easily accessible subtype of expanded fetal placental stromal cells as a simple therapeutic strategy for IBD and other similar major inflammatory diseases. Furthermore, they provide a solid basis to pave the way for more in-depth investigations into this innovative approach.

We propose that future studies should further explore the effect of the f-hPSCs effects with additional treatment arms and a higher number of test animals in order to better elucidate the underlying mechanisms involved in their effect. We suggest that further detailed studies, with more experimental arms and a higher number of animals, should strengthen the conclusions from our study on the efficacy of f-hPSCs injections in safely mitigating IBD and similar inflammatory diseases.

The safety of the clinical application of our findings is encouraged by a recently published ongoing clinical study on the safety of using commercially produced PLX-R18 cells [56]. This clinical study was inspired by our preliminary preclinical studies using their commercially produced PLX-18 cells (with a similar phenotype to our f-hPSCs) for enhancing bone marrow regeneration with both our own cells and the commercial cell sources [16,19]. This ongoing study has demonstrated the safety of the injection of these cells, which may be relevant to the potential future clinical application of f-hPSCs for the treatment of CD.

## 4. Methods and Materials

### 4.1. The Mouse Model

C57BL/6JOla black mice, 8–9 weeks old, were used as a model for induced IBD. The mice were purchased from Harlan/Envigo-RMS Ltd., Jerusalem, Israel (ISO 9001:2015). The animal experiments were planned and performed to conform to the guidelines of the Hebrew-University-Hadassah Medical Center Institutional Animal Welfare Committee for this study (#MD-15-14388-5). The DSS-induced CD mouse model was chosen because cryptitis and crypt depletion, common features of IBD in humans, were also observed in these mice following DSS-induced colitis [32,33]. Though both males and females develop robust DSS-induced IBD by this procedure, the male mice seem to develop a more rapidly aggressive disease than females; therefore, male mice were chosen. Four experimental groups were tested. IBD was induced in three of the groups by DSS exposures, one group of 12 mice was not treated for IBD, 11 mice exposed to DSS were treated IP with f-hPSCs, and an additional small control group of 6 mice were treated IM with the f-hPSC. The experimental system was tested prior to the experiment for dose/complication occurrence to set up the protocol with no loss of animals and verification of no complication of the injection of the cells as previously reported for IM delivery.

The mice in this study were housed in large filter-covered boxes, with 6–8 mice per box, in a strictly maintained Specific Pathogen-Free (SPF) animal facility. The experiment began following a one-week acclimatization period following the randomization of the mice into the different groups tested. Each mouse was identified with a tag and monitored five days a week for body weight and general condition. The DSS dosing and procedures were pre-tested to ensure the survival of all mice throughout the follow-up period in all study arms. The average initial weight of the mice in the tested groups before the onset of the experiment ranged from 20.0 to 20.3 g. Weight changes for each animal were recorded as a percentage of its initial weight, and the average weight of each group was tracked throughout the follow-up period.

### 4.2. f-hPSCs Preparation and Their Cell Surface Phenotype

The f-hPSCs were isolated from placentae of planned cesarean sections with male offspring which were donated with informed parental consent and approval from the Institutional Helsinki Committee for the use of human-derived materials (#0361-14HMO). The fetal placental connective tissues were dissected and f-hPSCs were isolated and expanded in vitro as previously described in detail [16,18]. Only expanded f-hPSCs populations from the fetal placenta tissue of a male offspring were used for f-hPSCs isolation. The expanded f-hPSCs were confirmed to be 100% of male placenta tissue by their pure X-Y karyotype by FISH assay. The surface markers of the f-hPSCs were determined as previously described [16,17,18,19] and the gating of the positive cells was based on the reading of relevant isotype controls (Figure 1A).

### 4.3. The Induction of IBD by DSS in Drinking Water 

The mice were induced to develop IBD by exposing them to 3.5% DSS in their drinking water. To examine the impact of f-hPSCs on the inflammatory and regenerative processes in the intestine, four experimental groups were studied: three groups received 3.5% DSS in water for 4 days, while one group served as the control group. On days 4 and 6, the f-hPSCs were administered either intramuscularly (IM) or intra-peritoneally (IP) to the treated groups exposed to DSS, while no cells were injected into the non-cell-treated control group. In the case of apparent signs of dehydration, the mice were injected subcutaneously in the abdomen with 1 mL of normal saline, and wetted food was placed in their cages to prevent possible fatal effects of the DSS. All mice were eventually euthanized at the end of the follow-up, on day 16 from the onset of the experiment.

### 4.4. f-hPSCs Injections

The f-hPSCs (2 × 10^6^ cells) for IP injection on days 4 and 6 of the follow-up were prepared by suspending the cells in 0.2 mL Plasmalite buffered solution. An additional small control group was injected IM on days 4 and 6 with the same total number of cells, suspended in 0.1 mL Plasmalite, which were injected into the large thigh muscles of both legs, as previously described [12]. Since we suspected that one boost of f-hPSCs may not be sufficient, in order to maintain the therapeutic effect, an additional injection of f-hPSCs was delivered 4 days after the first injection, based on our experience in previous studies with f-hPSCs treatments [16,17]. The optimal cell number delivery associated with no apparent local reaction is based on previous experimental work in which those cells were successfully tested in IM injection in mice model for the study of their pro-regenerative and anti-inflammatory effect [17,20,21].

### 4.5. Termination of the Experiments and Samples Collection

The mice were euthanized at the end of the experiment by overdose injection IP of 10 mg Ketamine in 0.2 mL normal saline and 1 mg Xylazine. Blood samples for CBC analyses were immediately drawn by a fine syringe from the still-beating hearts of the heavily anesthetized mice into dedicated blood collection micro-tubes containing EDTA. Then, the colon was dissected, rinsed, and measured. A short colon sample adjacent to the cecum was dissected and processed for histological analysis. This included histology with H&E staining and immune-histochemistry of macrophages staining with CD68-horseradish peroxidase (HRP), as detailed below.

Complete blood count (CBC) analysis was performed using the BC-2800Vet Mindray Hematology Analyzer (Guangzhou, China), calibrated specifically for differential blood counts in mice.

### 4.6. Assessment of Inflammatory Process by MLN Cell Count and WBC Cellularity

The MLN cells were collected from the sacrificed mice, and transferred to low-glucose DMEM. Then, the tissue samples were fully smashed by a plastic syringe and passed through a mesh. The collected cells were subsequently re-suspended in DMEM and counted by Countess II Cell Counter (Life Technologies, Thermo-Fisher, Waltham, MA, USA).

### 4.7. Assessment of the Severity of Inflammatory Processes by Neutrophil Activity by MPO Activity Assay in the Colon Samples

The colons collected from the euthanized mice were washed and their content was thoroughly rinsed with normal saline solution using Venflon tubing (Becton, Dickinson LTD, Wokingham Berkshire, UK).

The cleansed colons were then weighted and their length measured. A short colon sample of ~1 mm, adjacent to the caecum, was cut off and fixed for histological analysis. The rest of the colons were homogenized for 5 min on ice in 1 mL of 0.5% hexadecyltrimethylammonium bromide (HATB, #SLBK3798V) (Sigma-Aldrich, Science Park, Rehovot, Israel) using a blade homogenizer. The homogenized tissue was collected into small tubes and subjected to freeze and thaw cycles twice. The single cells in the test tubes were further dissociated by cycles of 15 s. high-power ultra-sonification. Then, the samples were centrifuged (13,000× *g*) for 15 min at ~4 °C and the supernatant was collected. In total, 10 µL of the supernatant from each sample was taken into 96 wells plate. Calibration solutions and samples were added to the 96-well plate. Then, 290 µL of O-dianisidine (Sigma-Aldrich Lot #MKBP7560V) was added to each well for initiation of the MPO assay. The OD of the samples was read immediately with Sunrise Tecan (Männedorf, Switzerland) plate reader at 460 nm. The results (in unit/mg) were normalized to the initial weight of the samples.

### 4.8. Colon Histology for Evaluation of Inflammatory Process

The colon tissue samples for histology were fixed at room temperature for 24 h in 4% formaldehyde and then transferred to 70% ethanol until their further processing. The fixed samples were embedded in paraffin, cut into 4 µm sections, and stained by H&E.

Immuno-histochemical HRP-CD68 staining to assay tissue macrophage (Mϕ) density was performed on 4 µm sections. The cell number per section area was counted by Leica Bond III system as follows: the slides were pretreated with epitope-retrieval solution for 10 min (ER2, Leica Biosystems Ltd., Newcastle, UK), followed by 30 min incubation with rabbit anti CD68 primary antibody (ab125212), 1:1500 (Abcam, Cambridge, UK), Leica Refine-HRP kit (Leica Biosystems Ltd, Newcastle., UK), before counter-staining with Hematoxylin. The histological samples were photographed sequentially at various magnifications. These full-section photographs were subsequently utilized for quantitative assessment of Mϕ prevalence and for objective, blinded pathological expert scoring. For the presentation of the full tissue sections, the histological samples on the slides were photographed in sequential pictures at different magnifications. For the presentation of a conclusive view of the full area of the gut cross-section, a series of sequential photographs of medium magnification (mag × 100–200) were taken. The series of such pictures of the same magnification were combined and stitched together by the photo-editing program to yield a high-resolution large photographic overview of a whole area of the gut section, from which specific regions of interest were presented with different magnifications in the results.

The prevalence of immune HRP-CD68 Mϕ was evaluated accurately by automatic counting of brown-stained cells per tissue section stained with anti-CD68 conjugated with HRP as analyzed with Nikon Element software version 6.10.01 (NIS) (Nikon, Melvile, NY, USA), of stained cells in stained sections. The dark brown-stained cells were identified in the tissue section areas within the slide. The results are presented as the number of cells per area of the tissue section of interest.

### 4.9. Statistics

All graph designs and statistical analyses were conducted with the aid of Microsoft Excel’s statistical software package. Statistical significance assessments were carried out with the use of standard paired Student’s *t*-test to compare the results among different experimental arms and presented as mean ± standard errors (SDs).

## Data Availability

Related data available upon request to R.G.

## References

[1] Hold G.L., Smith M., Grange C., Watt E.R., El-Omar E.M., Mukhopadhya I. (2014). Role of the gut microbiota in inflammatory bowel disease pathogenesis: What have we learnt in the past 10 years?. World J. Gastroenterol..

[2] Strober W. (2013). Impact of the gut microbiome on mucosal inflammation. Trends Immunol..

[3] Bandzar S., Gupta S., Platt M.O. (2013). Crohn’s disease: A review of treatment options and current research. Cell. Immunol..

[4] Singh S., Andersen N.N., Andersson M., Loftus E.V., Jess T. (2018). Comparison of infliximab with adalimumab in 827 biologic-naive patients with Crohn’s disease: A population-based Danish cohort study. Aliment. Pharmacol. Ther..

[5] Feagan B.G., Rutgeerts P., Sands B.E., Hanauer S., Colombel J.F., Sandborn W.J., Van Assche G., Axler J., Kim H.J., Danese S. (2013). Vedolizumab as induction and maintenance therapy for ulcerative colitis. N. Engl. J. Med..

[6] Sands B.E., Feagan B.G., Peyrin-Biroulet L., Danese S., Rubin D.T., Luo A., Nguyen D.D., Lu J., Yen M., Leszczyszyn J. (2024). Phase 2 trial of anti-TL1A monoclonal antibody Tulisokibart for ulcerative colitis. N. Engl. J. Med..

[7] Mishra R., Dhawan P., Srivastava A.S., Singh A.B. (2020). Inflammatory bowel disease: Therapeutic limitations and prospective of the stem cell therapy. World J. Stem Cells.

[8] Mayer L., Pandak W.M., Melmed G.Y., Hanauer S.B., Johnson K., Payne D., Faleck H., Hariri R.J., Fischkoff S.A. (2013). Safety and tolerability of human placenta-derived cells (PDA001) in treatment-resistant crohn’s disease: A phase 1 study. Inflamm. Bowel Dis..

[9] Pak S., Hwang S.W., Shim I.K., Bae S.M., Ryu Y.M., Kim H.B., Do E.J., Son H.N., Choi E.J., Park S.H. (2018). Endoscopic transplantation of mesenchymal stem cell sheets in experimental colitis in rats. Sci. Rep..

[10] Liao Y., Lei J., Liu M., Lin W., Hong D., Tuo Y., Jiang M.H., Xia H., Wang M., Huang W. (2016). Mesenchymal stromal cells mitigate experimental colitis via insulin-like growth factor binding protein 7-mediated Immunosuppression. Mol. Ther. J. Am. Soc. Gene Ther..

[11] Trebol J., Georgiev-Hristov T., Pascual-Miguelanez I., Guadalajara H., Garcia-Arranz M., Garcia-Olmo D. (2022). Stem cell therapy applied for digestive anastomosis: Current state and future perspectives. World J. Stem Cells.

[12] El-Nakeep S. (2022). Stem cell therapy for the treatment of Crohn’s disease; Current obstacles and future hopes. Curr. Stem Cell Res. Ther..

[13] Melmed G.Y., Pandak W.M., Casey K., Abraham B., Valentine J., Schwartz D., Awais D., Bassan I., Lichtiger S., Sands B. (2015). Human placenta-derived cells (PDA-001) for the treatment of moderate-to-severe Crohn’s Disease: A Phase 1b/2a Study, *Inflamm*. Bowel Dis..

[14] Caplan A.I. (2017). Mesenchymal Stem Cells: Time to change the name!. Stem Cells Transl. Med..

[15] Lee R.H., Pulin A.A., Seo M.J., Kota D.J., Ylostalo J., Larson B.L., Semprun-Prieto L., Delafontaine P., Prockop D.J. (2009). Intravenous hMSCs improve myocardial infarction in mice because cells embolized in lung are activated to secrete the anti-inflammatory protein TSG-6. Cell Stem Cell.

[16] Gaberman E., Pinzur L., Levdansky L., Tsirlin M., Netzer N., Aberman Z., Gorodetsky R. (2013). Mitigation of Lethal Radiation Syndrome in Mice by Intramuscular Injection of 3D Cultured Adherent Human Placental Stromal Cells. PLoS ONE.

[17] Volinsky E., Lazmi-Hailu A., Cohen N., Adani B., Faroja M., Grunewald M., Gorodetsky R. (2020). Alleviation of acute radiation-induced bone marrow failure in mice with human fetal placental stromal cell therapy. Stem Cell Res Ther.

[18] Adani B., Sapir E., Volinsky E., Lazmi-Hailu A., Gorodetsky R. (2022). Alleviation of severe skin insults following high-dose irradiation with isolated human fetal placental stromal cells. Cryobiology.

[19] Pinzur L., Akyuez L., Levdansky L., Blumenfeld M., Volinsky E., Aberman Z., Reinke P., Ofir R., Volk H.D., Gorodetsky R. (2018). Rescue from lethal acute radiation syndrome (ARS) with severe weight loss by secretome of intramuscularly injected human placental stromal cells. J. Cachexia Sarcopenia Muscle.

[20] Amend B., Buttgereit L., Abruzzese T., Harland N., Abele H., Jakubowski P., Stenzl A., Gorodetsky R., Aicher W.K. (2023). Regulation of immune checkpoint antigen CD276 (B7-H3) on human placenta-derived mesenchymal stromal cells in GMP-compliant cell culture media. Int. J. Mol. Sci..

[21] Shapira I., Fainstein N., Tsirlin M., Stav I., Volinsky E., Moresi C., Ben-Hur T., Gorodetsky R. (2016). Placental stromal cell therapy for experimental autoimmune encephalomyelitis: The role of route of cell delivery. Stem Cells Transl. Med..

[22] Pampalone M., Corrao S., Amico G., Vitale G., Alduino R., Conaldi P.G., Pietrosi G. (2021). Human amnion-derived mesenchymal stromal cells in cirrhotic patients with refractory ascites: A possible anti-inflammatory therapy for preventing spontaneous bacterial Peritonitis. Stem Cell Rev. Rep..

[23] Abumaree M.H., Al Jumah M.A., Kalionis B., Jawdat D., Al Khaldi A., AlTalabani A.A., Knawy B.A. (2013). Phenotypic and functional characterization of mesenchymal stem cells from chorionic villi of human term placenta. Stem Cell Rev..

[24] Duan L., Huang H., Zhao X., Zhou M., Chen S., Wang C., Han Z., Han Z.C., Guo Z., Li Z. (2020). Extracellular vesicles derived from human placental mesenchymal stem cells alleviate experimental colitis in mice by inhibiting inflammation and oxidative stress. Int. J. Mol. Med..

[25] Eiro N., Fraile M., Gonzalez-Jubete A., Gonzalez L.O., Vizoso F.J. (2022). Mesenchymal (Stem) stromal Cells based as new therapeutic alternative in inflammatory bowel disease: Basic mechanisms. experimental and clinical evidence and challenges. Int. J. Mol. Sci..

[26] Fu Y., Zhang C., Xie H., Wu Z., Tao Y., Wang Z., Gu M., Wei P., Lin S., Li R. (2023). Human umbilical cord mesenchymal stem cells alleviated TNBS-induced colitis in mice by restoring the balance of intestinal microbes and immunoregulation. Life Sci..

[27] Ke C., Biao H., Qianqian L., Yunwei S., Xiaohua J. (2015). Mesenchymal stem cell therapy for inflammatory bowel diseases: Promise and challenge. Curr. Stem Cell Res. Ther..

[28] Lightner A.L., Irving P.M., Lord G.M., Betancourt A. (2024). Stem cells and stem cell-derived factors for the treatment of inflammatory bowel disease with a particular focus on perianal fistulizing disease: A Minireview on future perspectives. BioDrugs.

[29] Cao X., Duan L., Hou H., Liu Y., Chen S., Zhang S., Liu Y., Wang C., Qi X., Liu N. (2020). IGF-1C hydrogel improves the therapeutic effects of MSCs on colitis in mice through PGE(2)-mediated M2 macrophage polarization. Theranostics.

[30] Bai K., Li X., Zhong J., Ng E.H.Y., Yeung W.S.B., Lee C.L., Chiu P.C.N. (2021). Placenta-derived exosomes as a modulator in maternal immune tolerance during pregnancy. Front. Immunol..

[31] Sendon-Lago J., Rio L.G., Eiro N., Diaz-Rodriguez P., Avila L., Gonzalez L.O., Vizoso F.J., Perez-Fernandez R., Landin M. (2021). Tailored hydrogels as delivery platforms for conditioned medium from mesenchymal stem cells in a model of acute colitis in mice. Pharmaceutics.

[32] Perse M., Cerar A. (2012). Dextran sodium sulphate colitis mouse model: Traps and tricks. J. Biomed. Biotechnol..

[33] Chassaing B., Aitken J.D., Malleshappa M., Vijay-Kumar M. (2014). Dextran sulfate sodium (DSS)-induced colitis in mice. Curr. Protoc. Immunol..

[34] Galvez J. (2014). Role of Th17 cells in the pathogenesis of human IBD. ISRN Inflamm..

[35] Abomaray F.M., Al Jumah M.A., Alsaad K.O., Jawdat D., Al Khaldi A., AlAskar A.S., Al Harthy S., Al Subayyil A.M., Khatlani T., Alawad A.O. (2016). Phenotypic and functional characterization of mesenchymal stem/multipotent stromal cells from Decidua Basalis of human term placenta. Stem Cells Int..

[36] Yi Q., Wang J., Song Y., Guo Z., Lei S., Yang X., Li L., Gao C., Zhou Z. (2019). Ascl2 facilitates IL-10 production in Th17 cells to restrain their pathogenicity in inflammatory bowel disease. Biochem. Biophys. Res. Commun..

[37] Riddell M.R., Winkler-Lowen B., Chakrabarti S., Dunk C., Davidge S.T., Guilbert L.J. (2012). The characterization of fibrocyte-like cells: A novel fibroblastic cell of the placenta. Placenta.

[38] Zhao Y., Gillen J.R., Harris D.A., Kron I.L., Murphy M.P., Lau C.L. (2014). Treatment with placenta-derived mesenchymal stem cells mitigates development of bronchiolitis obliterans in a murine model. J. Thorac. Cardiovasc. Surg..

[39] Lahiani A., Zahavi E., Netzer N., Ofir R., Pinzur L., Raveh S., Arien-Zakay H., Yavin E., Lazarovici P. (2015). Human placental eXpanded (PLX) mesenchymal-like adherent stromal cells confer neuroprotection to nerve growth factor (NGF)-differentiated PC12 cells exposed to ischemia by secretion of IL-6 and VEGF. Biochim. Biophys. Acta.

[40] Petrou P., Gothelf Y., Argov Z., Gotkine M., Levy Y.S., Kassis I., Vaknin-Dembinsky A., Ben-Hur T., Offen D., Abramsky O. (2016). Safety and clinical effects of mesenchymal stem cells secreting neurotrophic factor transplantation in patients with amyotrophic lateral Sclerosis: Results of Phase 1/2 and 2a Clinical Trials. JAMA Neurol..

[41] Adams K.M., Yan Z., Stevens A.M., Nelson J.L. (2007). The changing maternal “self” hypothesis: A mechanism for maternal tolerance of the fetus. Placenta.

[42] Chang C.J., Yen M.L., Chen Y.C., Chien C.C., Huang H.I., Bai C.H., Yen B.L. (2006). Placenta-derived multipotent cells exhibit immunosuppressive properties that are enhanced in the presence of interferon-gamma. Stem Cells.

[43] Gorodetsky R., Aicher W.K. (2021). Allogenic use of human placenta-derived stromal cells as a highly active subtype of mesenchymal stromal cells for cell-based therapies. Int. J. Mol. Sci..

[44] Maier C.L., Pober J.S. (2011). Human placental pericytes poorly stimulate and actively regulate allogeneic CD4 T cell responses. Arterioscler. Thromb. Vasc. Biol..

[45] Macholdova K., Machackova E., Proskova V., Hromadnikova I., Klubal R. (2019). Latest findings on the placenta from the point of view of immunology. tolerance and mesenchymal stem cells, Ceska gynekologie/Ceska lekarska spolecnost. J. Ev. Purkyne.

[46] Forbes G.M. (2017). Mesenchymal Stromal Cell Therapy in Crohn’s Disease. Dig Dis.

[47] Magatti M., De Munari S., Vertua E., Gibelli L., Wengler G.S., Parolini O. (2008). Human amnion mesenchyme harbors cells with allogeneic T-cell suppression and stimulation capabilities. Stem Cells.

[48] Wang Y., Zhao X., Li Z., Wang W., Jiang Y., Zhang H., Liu X., Ren Y., Xu X., Hu X. (2024). Decidual natural killer cells dysfunction is caused by IDO downregulation in dMDSCs with Toxoplasma gondii infection. Commun Biol.

[49] Kshirsagar S.K., Alam S.M., Jasti S., Hodes H., Nauser T., Gilliam M., Billstrand C., Hunt J.S., Petroff M.G. (2012). Immunomodulatory molecules are released from the first trimester and term placenta via exosomes. Placenta.

[50] Chen X., Zhao X., Wang H., Yang Z., Li J., Suo H. (2017). Prevent Effects of Lactobacillus Fermentum HY01 on Dextran Sulfate Sodium-Induced Colitis in Mice. Nutrients.

[51] Nordgren S., McPheeters G., Svaninger G., Öresland T., HulténInt L. (1997). Small bowel length in inflammatory bowel disease. J. Colorect. Dis..

[52] Sydora B.C., Albert E.J., Foshaug R.R., Doyle J.S., Churchill T.A., Fedorak R.N. (2012). Intravenous injection of endogenous microbial components abrogates DSS-induced colitis. Dig. Dis. Sci..

[53] Li C., Zhang W., Jiang X., Mao N. (2007). Human-placenta-derived mesenchymal stem cells inhibit proliferation and function of allogeneic immune cells. Cell Tissue Res..

[54] Liu W., Morschauser A., Zhang X., Lu X., Gleason J., He S., Chen H.J., Jankovic V., Ye Q., Labazzo K. (2014). Human placenta-derived adherent cells induce tolerogenic immune responses. Clin. Transl. Immunol..

[55] Ouyang Y., Mouillet J.F., Coyne C.B., Sadovsky Y. (2014). Review: Placenta-specific microRNAs in exosomes—Good things come in nano-packages. Placenta.

[56] McGuirk J.P., Metheny L., Pineiro L., Litzow M., Rowley S.D., Avni B., Tamari R., Lazarus H.M., Rowe J.M., Sheleg M. (2023). Placental expanded mesenchymal-like cells (PLX-R18) for poor graft function after hematopoietic cell transplantation: A phase I study. Bone Marrow Transpl..

